# Rheumatoid Arthritis-associated Interstitial Lung Disease in Greece: A Multicentre Epidemiological and Clinical Study

**DOI:** 10.31138/mjr.29.4.236

**Published:** 2018-12-18

**Authors:** Theofanis Karageorgas, Prodromos Sidiropoulos, Dimitrios Vassilopoulos, Dimitrios Boumpas

**Affiliations:** 1 Joint Rheumatology Program, Clinical Immunology-Rheumatology Unit, 4^th^ Department of Medicine, National and Kapodistrian University of Athens-School of Medicine, Attikon General Hospital, Athens, Greece,; 2 Joint Rheumatology Program, Rheumatology and Clinical Immunology Department, University of Crete-School of Medicine, University Hospital of Heraklion, Crete, Greece,; 3 Joint Rheumatology Program, Clinical Immunology-Rheumatology Unit, 2^nd^ Department of Medicine and Laboratory, National and Kapodistrian University of Athens-School of Medicine, Hippokration General Hospital, Athens, Greece

**Keywords:** Rheumatoid Arthritis, Interstitial Lung Disease, DMARDs

## Abstract

Interstitial Lung Disease (ILD) represents one of the most severe complications of Rheumatoid Arthritis (RA). Preliminary data from the RA Greek cohort show a prevalence of 5.3%. Due to scarcity of data, little is known regarding the epidemiological and clinical features of Greek patients with RA-ILD. Moreover, use of pulmonary function tests for prognostic purposes in patients with RA-ILD is still not sufficiently studied. Interestingly, the treatment approach of patients with RA-ILD remains controversial due to high risk of infection, possible drug-related pulmonary toxicity, and scarce evidence regarding the efficacy of medications used in these patients. The aim of this research protocol is to collect data from patients with RA-ILD followed in multiple centres across Greece in order to identify the clinical and epidemiological features of these patients. The second part of the study focuses on the prospective data collection regarding the progression of ILD, the response to different treatment modalities and the incidence of adverse events attributed either to the disease itself or to its treatment in patients with RA-ILD. This study may provide useful evidence in exploring both the natural history and the risk factors contributing to the development of ILD, as well as the efficacy and the adverse events attributed to the medications used in Greek patients with RA-ILD; thus ameliorating the therapeutic approach of RA-ILD patients in daily clinical practice.

## INTRODUCTION

Interstitial Lung Disease (ILD) represents one of the most severe complications of Rheumatoid Arthritis (RA). Epidemiological data from Mayo Clinic reveal that ILD development in RA patients has a hazard ratio (HR) of 8.96 which is further heightened in patients with increased age, male gender, smokers and with high disease burden (high ESR, HAQ, RF and ACPA titers).^1^ Preliminary data from the “Attikon” University Hospital RA-ILD cohort show that 31% of the patients are male, 56% are seropositive for RF and/or ACPA, and 44% have radiographic erosions. ILD prevalence in RA patients from a multicentre study involving 15 European countries was reported at 4.5%,^2^ and preliminary data from the RA Greek cohort show a prevalence of 5.3%. Interestingly, a recent large multicentre UK study reported that in 83% of RA patients arthritis preceded the diagnosis of ILD while in the remaining 17% of patients arthritis appeared synchronously (7%) or metachronously (10%) of the ILD diagnosis.^3^ Importantly, mortality risk is 3 times higher in RA patients with ILD compared with RA patients without ILD while mean survival since the diagnosis of symptomatic ILD ranges from 2–6 years. Overall, ILD confers 13% of the increased mortality risk noted in RA patients compared to the general population.^1^

Recent data highlight the crucial prognostic role of the ILD HRCT pattern. Thus, the Usual Interstitial Pneumonia (UIP) pattern is discovered with increased frequency in RA patients and is associated with significantly worse survival compared to other HRCT patterns (NSIP, OP, Overlap).^4,5^ To our knowledge, no study so far exploring the prevalence of the different HRCT patterns in Greek RA patients has been published.

Moreover, studies in Systemic Sclerosis demonstrate that pulmonary function tests play a crucial prognostic role in assessing both the severity as well as the progression of ILD in these patients.^6^ Reduction of FVC and/or DLCO levels below of 70% of predicted values is significantly associated both with ILD progression and ILD-related mortality in systemic sclerosis patients. Similar data regarding RA-ILD patients are scarce.^7^ Consequently, use of such PFTs parameters in the assessment and management of RA-ILD patients in clinical practice is not evidence-based.

Whilst all other extra-articular manifestations of RA show decreasing frequency over the years mainly due to the use of highly-effective DMARDs, RA-ILD frequency is rising; demonstrating that its management remains highly controversial. Available DMARDs used in RA management (e.g. Methotrexate, TNFi, etc.) have been invariably associated with de novo detection or deterioration of pre-existing ILD as well as other pulmonary manifestations.^8–10^ Observational studies in RA-ILD patients have also established the increased risk for infections (both common and opportunistic) due to the use of immunomodulatory/immunosuppressive medications.^11,12^ Further increasing the uncertainty regarding management decisions in RAILD patients, the effectiveness of DMARDs in treating or at least halting the progression of ILD is supported by weak evidence mainly by non-randomized small sample sized studies^13^ or case series.^14,15^

## STUDY DESIGN

Detailed data of RA patients (fulfilling the 2010 ACR/EULAR criteria)^16^ with and without ILD who are referred and followed at the participating hospitals will be collected (both in hardcopy datasheets and electronic database). For each patient the initial dataset includes:

- Demographical data (age, gender, profession, etc.);
- Comorbidities (Diabetes Mellitus, Hypertension, Dyslipidaemia, history of cardiovascular disease, BMI, smoking, history of COPD/asthma, thyroid disease, osteoporosis, history of neoplastic disease, HBV and HCV, history of tuberculosis PPD and/or IGRAs);
- Clinical and Laboratory data regarding RA (disease duration, extra-articular manifestations, radiographic evidence of erosions, RF and ACPA titers, ESR, CRP, etc.);
- Past and current medications use (relative to RA); and
- Vaccinations.

Chest HRCT will be adopted for establishing the diagnosis of ILD. Additional data regarding ILD will be collected including date of ILD diagnosis, presenting symptoms as well as the subjective respiratory functional status (as per MRC), HRCT pattern of ILD (classification in UIP, NSIP, OP, overlap/other) and the results of PFTs (FVC, FEV1, DLCO, TLC, both in % of predicted values and in L).

At the follow-up visits taking place every 3 months (until the completion of at least 1 year since the patient’s initial registration) the following data will be collected:

- Data regarding RA
  1. Disease activity assessment (DAS28, CDAI) and functional status (HAQ);
  2. Treatment and eventual treatment/dose modifications (csDMARDs, bDMARDs, corticosteroids); and
  3. Adverse events such as infections, hospitalizations or other events attributed either to the disease itself or to its treatment.
- Data regarding ILD
  1. Patient’s respiratory functional status (as per MRC);
  2. Results of PFTs (FVC, FEV1, DLCO, TLC, both in % of predicted values and in L); and
  3. HRCT report.

## METHODS

The first part consists of a cross-sectional study between patients with RA-ILD and RA-non ILD (gender- and age-matched control group) in order to identify independent risk factors associated with ILD and the relative risk attributed to each one of these risk factors.

The second part of the study focuses on the prospective data collection regarding RA-ILD patients aiming at the determination of the following outcomes:

1. Prevalence of the different HRCT patterns;
2. Severity assessment of ILD in the initial and successive follow-up visits;
3. Response to different treatment modalities; and
4. Incidence of adverse events attributed either to the disease itself or to its treatment.

Our goal is to collect data from at least 100 RA-ILD patients for this study to be sufficiently powered. Moreover, we aim to complete both data collection and statistical analysis by July 2019 in order to publish the results in the forthcoming 2019 ACR Congress and to peer-reviewed international journals.

### Statistical analysis

All analyses will be performed with the use of Microsoft Excel 2013 and IBM SPSS Statistics v.20 software. At first, data will be analysed by descriptive statistics. Continuous variables will be presented by mean and median values and standard deviations. Categorical variables will be presented by percentages. Multivariate analysis will be performed in order for independent risk factors to be assessed.

## ANTICIPATED BENEFITS

This is the first study aiming to collect data from RA-ILD patients followed in multiple hospital centres in Greece in order to assess both the natural history and the risk factors contributing to the development of ILD. Exploring both the efficacy and the adverse events attributed to the medications used in patients with RA-ILD will provide useful evidence leading to the amelioration of the therapeutic approach to RA-ILD patients in daily clinical practice. An additional benefit of this study will be the generation of a biobank of serum and nucleic acids for future biomarker studies.
